# Asymmetry of eye color in the common cuckoo

**DOI:** 10.1038/s41598-017-08071-1

**Published:** 2017-08-08

**Authors:** Ha-Na Yoo, Jin-Won Lee, Jeong-Chil Yoo

**Affiliations:** Korea Institute of Ornithology & Department of Biology, Kyung Hee University, Seoul, 02447 Republic of Korea

## Abstract

Bilateral symmetry is assumed to contribute to the evolution of eye color, with the left and right eye being the same color in most vertebrates; yet, few studies tested this assumption. Here, we compared the amount of iris flecking (black spots presented on the iris) between the left and right eye of 76 adult common cuckoos *Cuculus canorus*. We found considerable variation in the total amount of iris flecking among individuals, with variation being associated with body size and sex. We also found that the amount of iris flecking differed between the left and right eye and that this left-right asymmetry was not random, with the left eye almost always being darker than the right eye. Furthermore, this asymmetry was negatively associated with wing length; however, this effect was limited to individuals with dark eyes. Overall, the asymmetric, but non-random, distribution of iris flecking between the left and right eye may indicate that selection pressures driving asymmetry (such as visual lateralization) act on the development of iris colors, even though this effect might be limited, due to the role of bilateral symmetry.

## Introduction

The iris is a set of muscle that regulate pupil size, and exhibits remarkable variation in color, ranging from dark to bright, within and across vertebrates species^1–9^. In many species, not only the background color but also patterning (generated by the presence of spots and lines around the pupil) of the iris differ among individuals^10–12^. Over the last century, this variation in iris color has been extensively described in the published literature^13–16^, improving our understanding of its underlying mechanism and biological significance. Thus, it is now established that this variation in iris color is caused by the presence and absence of different types of pigmentation such as melanin, pteridines and purines, as well as superficial blood vessels and/or eye structure, irrespective of pigmentation^4, 9, 17^. However, the mechanisms involved in generating this variability seem to differ across species^4, 5^. In addition, the intra-specific variation of iris color is often associated with age, sex, social dominance, season and geography^2, 8, 11, 17, 18^. Consequently, iris color might provide information about the holder. For example, each human has a unique iris, in terms of colors and patterns, allowing it to be used to identify individuals (like fingerprinting)^19–21^. Yet, to date, most studies have focused on the color of just one eye, despite all vertebrates having two eyes. Furthermore few studies have compared both eyes with respect to iris color evolution, except some reports on abnormal heterochromia (multi-colored) irises^22^.

Anatomically, the iris may act as a barrier between the inside and outside of the eye. Thus, different selection pressures might influence the evolution of iris color on either side of the iris. Furthermore, these pressures might operate differently between the left and right eye. As a pair of external phenotypes that all vertebrates have, it is generally assumed that iris color has evolved under the selection of bilateral symmetry, which is one of the universal phenomena observed in the morphology of diverse living organisms^23^. Although this assumption does not always hold true^24, 25^, many studies have found that the degree of symmetry is an indicator of the phenotypic and/or genetic qualities of the holder, thereby playing an important role in sexual and natural selection^26–28^. For example, symmetry in the wings and tail of birds is selected for to increase aerodynamic efficiency. Consequently, species relying heavily on flight might have more symmetric wings and tails than species that rely less on flight^29^. Furthermore, the symmetry of the human face is associated with facial attractiveness to the opposite sex, making it important for mate selection^30^. However, the role of bilateral symmetry in the evolution of iris colors remains poorly defined. It might be hypothesized that iris color is the same between the left and right eye, and at the population level, any changes in iris colors may occur randomly between the left and right eye. Furthermore, it might be hypothesized that individuals with more symmetric iris color are genetically or phenotypically superior to those with less symmetric iris color, leading to their being preferred in terms of sexual and natural selection. The fact that heterochromia irises are often associated with pathology and/or congenital syndromes might support this possibility^31^.

However, in visual system, functional asymmetry between the left and right eye (i.e., visual lateralization) is the widespread norm across animal taxa, from insects to human^32–36^. The increase in visual asymmetry appears to enhance cognitive ability, behavioral performance, and thus, the biological fitness of individuals^37^. Visual lateralization is achieved based on the developmental and anatomical asymmetry of the visual nervous system as well as the cerebral nervous system^38, 39^. These asymmetric variations in the nervous system might also generate, or be associated with, the asymmetric morphology of eyes. For example, Hart *et al*.^40^ showed the asymmetric variation of retinal morphology with respect to photoreceptor distribution in the European starling *Sturnus vulgaris*. However, our knowledge on whether such internal asymmetric variation is also reflected to external eye phenotypes such as iris color remains limited, despite its functional relevance to visual systems in terms of affecting behavioral response^41, 42^. More generally, it is unclear how the development of visual lateralization is associated with the role of bilateral symmetry in the development of iris color. As a link between the front and back of the eye, the iris might be the location where these two selection pressures conflict with each other. Consequently, the evolutionary pathway of iris color might be more complicated than we currently perceive.

In this study, we used the common cuckoo *Cuculus canorus*, a species of avian brood parasites, to explore whether iris color is asymmetric in a way that would be expected by visual lateralization or, alternatively, whether iris color exhibits bilateral symmetry. Specifically, we quantified and compared the iris colors of both eyes of the same individual, placing focus on the number of black spots on the iris (hereafter termed “iris flecking”). Compared to iris background color, iris flecks are easier to quantify objectively with photographs taken in the field. The iris background color of the common cuckoo changes with age from dark brown when at the nestling/juvenile stage to bright yellow in adults^43^, which is known to be a trait mimicking *Accipiter* hawks to facilitate access to host nests^44, 45^. Iris color also varies with respect to the amount of iris flecking, with some individuals having clear bright yellow irises without flecks whereas in some individuals iris flecks form even a thick and dark ring around the pupil. However, the extent to which iris flecking vary among individuals and between the left and right eye of the same individual has not been determined, along with the biological implication of these differences in the common cuckoo. To achieve our objectives, we first quantified the amount of iris flecking on the left and right eyes of the same individual, and determined the degree of its symmetry. We then examined how variation in the symmetry of iris flecking, as well as the total amount, is associated with different biological characteristics such as body size and sex.

## Results

### Asymmetric variation of iris flecking

**Figure 1:**
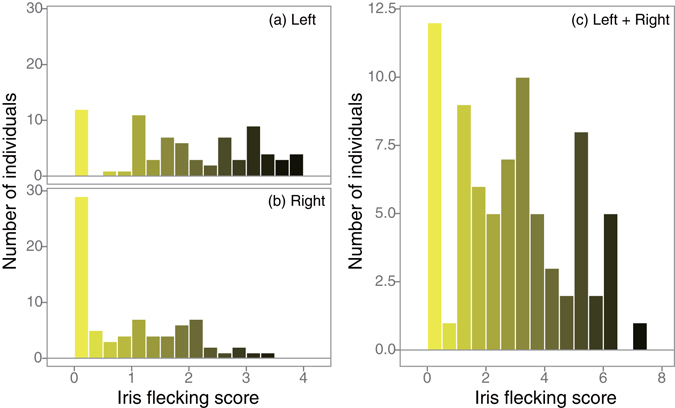
Frequency distribution of individuals according to the amount of iris flecking: (**a**) left eye, (**b**) right eye, and (**c**) both eye. The color was graded from bright yellow to black according to iris flecking score.

We found that total iris flecking varied considerably among individuals, from a score of 0 to over 6 (Fig. 1). Only 5 out of 76 birds had completely clear irises without any iris flecks in both eyes (score 0). Variation was not associated with capture-date (Pearson correlation: *r* = −0.12, *p* = 0.32), year (Kruskal-Wallis test: χ^2^ = 7.13, *df* = 3, *p* = 0.07) and site (*t*-test: *t* = 0.28, *p* = 0.78), and it was not skewed significantly to either side (skewness = 0.23; mean score = 2.89, median score = 2.90).

**Figure 2:**
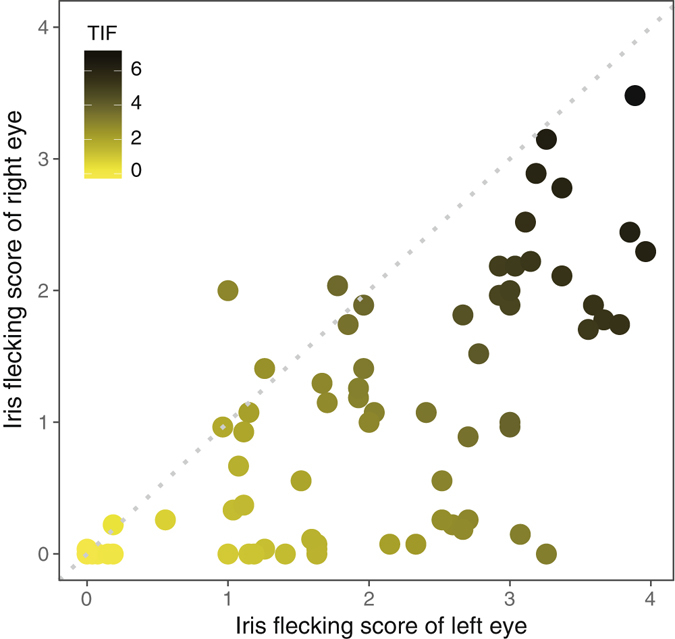
A scatter plot showing the relationship between the left and right eye in the amount of iris flecking. Each dot represents each individual and the color was graded according to total iris flecking (TIF). The dotted line indicates the location when the left and right eyes have the same amount of iris flecking.

**Figure 3:**
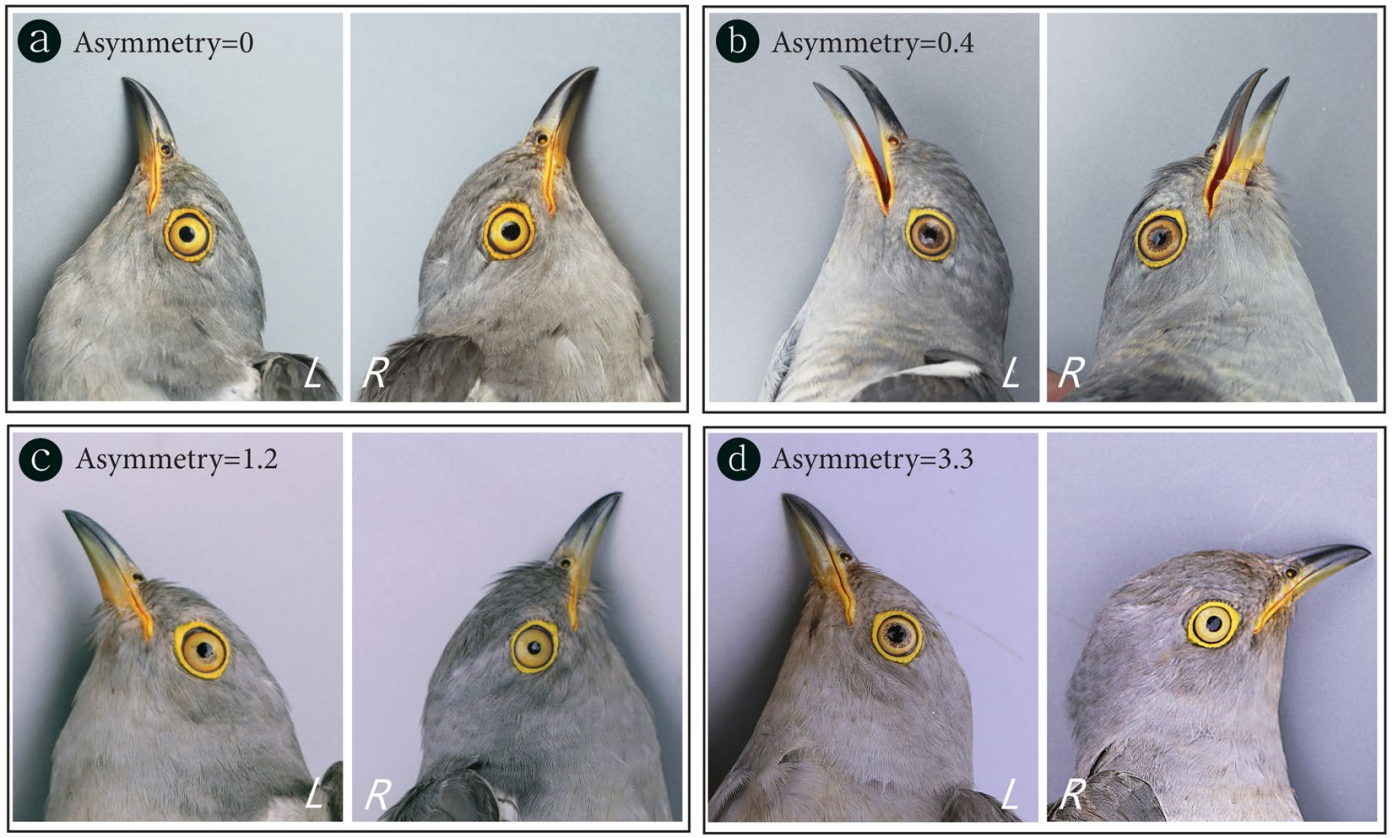
Photographs illustrating the variation of iris flecking asymmetry in the common cuckoo. Each set of photos shows the left and right side of the face of the same individual. ‘L’ and ‘R’ represent the left and right sides, respectively.

**Figure 4:**
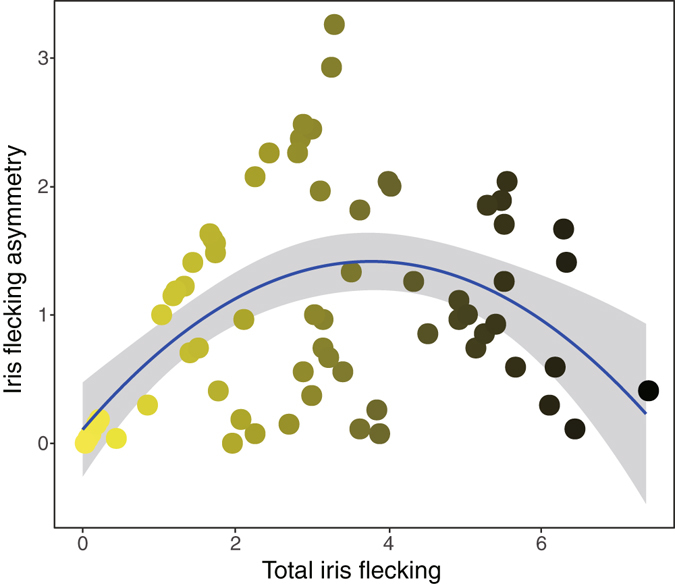
The variation of iris flecking asymmetry in relation to total iris flecking, and its quadratic fitted line (blue line) with 95% confidence interval (dark grey area). The equation for the model is y = −0.09x ^2^ + 0.69x + 0.11. Each dot represents each individual and the color was graded from yellow to black according to the total amount of iris flecking.

The overall amount of iris flecking was strongly correlated between the left and right eye (Spearman rank correlation: *r*_*s*_ = 0.73, *p* < 0.0001; Fig. 2) but it was not symmetric; only 6 out of 76 individuals had the exact same score between the left and right eye, whereas the eye scores of all other individuals differed (Figs 2 and 3). Interestingly, we found that the left eye was almost always darker than the right eye for individuals that had different scores between the both eyes (5 individuals had darker right eyes *vs*. 65 individuals had darker left eyes; Exact binomial test: *p* < 0.0001; Figs 3 and 4). There was no sexual difference in the extent of iris flecking asymmetry (median iris flecking: male = 1, female = 0.9; Wilcoxon rank sum test: *W* = 516, *p* = 0.86).

Iris flecking asymmetry had a significant quadratic relationship with total iris flecking with an increase in the difference as total iris flecking increased, followed by a decrease in the difference with further increase in total iris flecking (the quadratic term = −0.09, 95% CI: −0.13–−0.05, *p* < 0.0001; the linear term = 0.69, 95% CI: 0.43–0.96, *p* < 0.0001; Fig. 4). Likewise, inter-individual variation in iris flecking asymmetry showed a similar pattern with total iris flecking; the largest variation was observed at an iris flecking score of around 3 (Fig. 4).

### Biological significance of iris flecking

A general linear model showed a significant sexual difference in total iris flecking (Table 1), with males having a smaller amount of iris flecking than females (difference: 1.27, *p* = 0.02) and an almost significant relationship between total iris flecking and wing length (slope: −0.07, *p* = 0.057). However, their interaction was not significant (*p* = 0.19; Table 1), implying that the overall size-flecking relationship did not differ between sexes, although only males were significant in a simple correlation analysis (male: *r* = −0.30, n = 59, *p* = 0.02; female: *r* = −0.07, n = 17, *p* = 0.78).

**Table 1:** The summary of a general linear model regarding the effect of wing length and sex on total iris flecking in 76 common cuckoos.

Parameters	Estimate	95% CI	*p*
Wing length	−0.07	−0.15–0.002	0.057
Sex(male)	−1.27	−2.37–−0.18	0.02
Wing length: Sex	−0.1	−0.24–0.05	0.19

**Figure 5:**
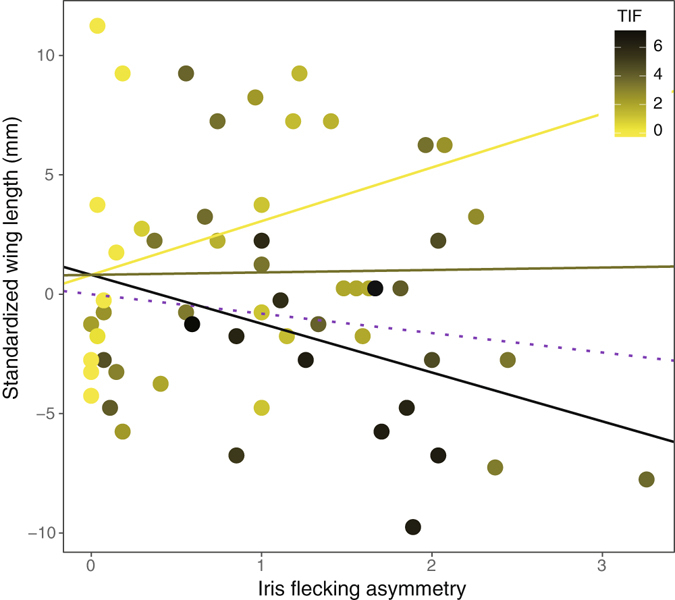
A figure illustrating the interaction effect between total iris flecking and iris flecking asymmetry on wing length in male common cuckoos. The grey line represents the expected slope (0.1) for individuals with mean iris flecking (2.50) while the yellow (slope = 2.25) and black (slope = −2.05) lines indicate the fitted line for individuals with clearer (mean iris flecking - one standard deviation, 0.68) and darker (mean iris flecking + one standard deviation, 4.32) iris. The dotted purple line (slope = −0.81) represents the fitted line for the univariate relationship between iris flecking asymmetry and wing length. Each dot represents each individual and the color was graded according to the total amount of iris flecking (TIF).

We analyzed whether asymmetry in iris flecking is associated with the variation in wing length for males that showed a significant correlation between total iris flecking and wing length. Although it was not statistically significant, there was an overall negative relationship between iris flecking asymmetry and wing length (Univariate linear regression: slope = −0.81, 95% CI: −2.37–0.74, *p* = 0.3, Fig. 5). However, because the degree of asymmetry was associated with total iris flecking, this phenomenon required analysis. In the model where total iris flecking was included as a covariate, we found a significant interaction effect of iris flecking asymmetry and total iris flecking on wing length (Interaction: estimate = −1.18, 95% CI: −2.18–−0.18, *p* = 0.02; Fig. 5). It indicates, for example, that iris flecking asymmetry almost had no effect on wing length (slope = 0.1, 95% CI: −1.55–1.76) for individuals with an average amount of iris flecking. However, for individuals having darker irises (total iris flecking: mean + 1 standard deviation), a negative effect was detected (slope: −2.05, 95% CI: −4.47–0.38). For individuals with clear irises (total iris flecking: mean – 1 standard deviation), the effect was positive (slope = 2.25, 95% CI: −0.23–4.49).

## Discussion

This study showed that the amount of iris flecking differed among individuals and, interestingly, between the left and right eyes of the same individual in the common cuckoo. This asymmetry in iris flecking was directional, with the left eye almost always being darker than the right eye. To the best of our knowledge, this study is the first to report non-random asymmetry in iris color between the left and right eye in a wild bird. It is difficult to explain this non-random variation but it might be expected if associated with asymmetric developments related to visual lateralization, rather than the selection of bilateral symmetry. It is well established that during the embryonic development of birds within the egg, the right eye receives more light stimulation than the left eye, due to asymmetry in the positioning of the embryo. This phenomenon ultimately triggers functional and anatomical asymmetry in the visual system, such as the tectofugal and thalamofugal systems^46–49^. Thus, as shown in pigeons and chickens, birds exhibit right-eye dominance with left-hemispheric dominance of the brain in visuomotor control^35^. Therefore, it could be hypothesized that asymmetric light stimulation during embryogenesis causes the directional asymmetry of iris color in the common cuckoo, due to the series of lateralized events described here. Consequently, some individuals with a darker right eye compared to left eye might be due to their being positioned incorrectly during the development in the egg (e.g., ‘head under left wing’, ^50^); however, this suggestion needs testing. Asymmetric iris flecking might aid the development process of visual lateralization by differentially regulating the amount of light that reaches the retina in the left and right eye. Dark irises transmit less light, which decreases sensitivity, but increases resolution. In contrast, bright irises function the other way round, which might ultimately generate different behavioral responses in terms of sensitivity and mobility^41, 42^. Thus, the development of different left-right iris coloration might be achieved in a way to enhance visual lateralization.

Alternatively, our results imply that the developmental schedule of iris colors is asymmetric between the left and right eyes, although the final color of the irises is the same. The presence of iris flecks might represent their age/developmental status, and might disappear after individuals have fully matured, as shown in some bird species with progressive changes in iris colors from dark to bright. For example, Sweijd and Craig^17^ showed age-related changes of iris colors in the African pied starling (*Spreo bicolor*). Specifically, juveniles had a dark brown iris, whereas adults had a creamy-white iris and subadults exhibited a variable amount of dark iris flecking with peripheral creamy-white in the iris. The authors showed that these variations occurred as a result of a progressive reduction of dark pigment in the anterior border layer of the iris, but did not compare the left and right eyes. Likewise, the non-random difference in iris flecking between the left and right eye in the common cuckoo might result from differences in the developmental process. The negative relationship between total iris flecking and body size might support this suggestion for the common cuckoo, given that they continue growing to a certain age, even after sexual maturity is reached. However, the amount of time for iris flecks to change or background color to appear seems to differ among species, ranging from a few months to several years^7, 14, 51^. Currently, it is unclear whether iris flecks are static or changeable in adult cuckoos, which needs to be resolved in future studies.

The question arises of why heterochromia irises are rarely observed in vertebrates, despite a potential relationship between iris color and asymmetric visual system. This phenomenon indicates that selection pressure derived from outside the eye for bilateral symmetry is stronger than that driven by visual asymmetry from inside the eye. This argument is based on the premise that symmetry in eye colors is important because it conveys information on the genetic and phenotypic qualities of the holder. The current study identified some relationship between iris flecking symmetry and wing length in males. Thus, the selection of bilateral symmetry might operate on the evolution of iris colors in the common cuckoo. However, the effect was limited, as symmetry in iris color affected the relationship with wing length differently with respect to the total amount of iris flecking. That is, individuals with more symmetric iris flecking tended to have longer wing lengths, but only among the individuals with dark eyes. Therefore, it remains unclear whether symmetry in iris flecking is related to individual quality, as predicted by the hypothesis of bilateral symmetry in this species; however this possibility should not be completely ruled out.

Another significant result that we found in this study was that females had darker eyes than males. It might imply either that the final amount of iris flecking differs between the sexes or it reflects the sexual difference in the development schedule of iris color. Irrespective of the underlying mechanism causing the difference, the dark eyes of females might help to facilitate access to host nests by decreasing conspicuousness and host aggression, at least in some parasite-host system. For example, Trnka *et al*.^45^ experimentally showed that the black-eyed dummy cuckoo received far less aggressive response than the dummy cuckoo with yellow eyes from the great reed warbler *Acrocephalus arundinaceus*, a host species breeding in dense reedbeds, and proposed that the host may primarily recognize the common cuckoos with their yellow eye around the nest. Nevertheless, it is unclear whether or not dark female eye is the adaptive trait that was acquired during the coevolutionary arms race against host aggression. Other species that have sexual differences in iris flecking also show that females have more iris flecking than males as shown in the common cuckoo^11^, and more generally, the iris color of males tend to change earlier than females in many species^14, 18^. Therefore, it might be useful to compare the sexual and species difference in the amount of iris flecking between the brood and non-brood parasites in the lineage of cuckoos to further elucidate the sexual difference in iris flecking and its adaptive significance with respect to brood parasitism.

Overall, our results showed that the presence of iris flecks was skewed to the left eye, possibly supporting that different iris colors and/or how iris color develops between the left and right eye is associated with selection pressures that favor asymmetry, such as visual lateralization, in the common cuckoo. However, at present, we do not know how iris flecks occur and how iris flecks are associated with visuomotor lateralization in this species. Additional behavioral/anatomical studies with captive populations would directly establish the relationship between iris flecks and developmental (a)symmetry in the common cuckoo. It is also interesting to determine if the similar pattern of iris flecking are also found in *Accipiter* hawks that the common cuckoo is hypothesized to mimic. Furthermore, the relative intensity of selection pressure on symmetric or asymmetric eye colors might differ among species, depending on whether the two eyes face forward (like humans) or laterally (like most fishes). In conclusion, a detailed comparison of left and right eye color across diverse animal taxa might broaden our understanding on the evolution of eye color in relation to visual lateralization and bilateral symmetry.

## Materials and Methods

### Fieldwork

Fieldwork to capture the common cuckoos was conducted at Jeollanam-do (34°48′N, 126°22′E) and Jeju-do (33°28′N, 126°49′E) in the Republic of Korea during the breeding seasons (May-July) from 2013 to 2016. In total, we captured 76 common cuckoos (males = 59, females = 17) using mist nets, with a decoy and playbacks, under the permission of local governments. Each individual was banded with a numbered metal ring, and the head-bill length, tarsus length, wing length (primary feathers), and weight were measured using standard methods^43^. Individuals were sexed by vocal cues in the field, with sex being subsequently confirmed by the molecular analysis of blood samples (20–30 μl) collected from the brachial vein of each individual^52^. Finally, we photographed the both sides of the face of each individual using a digital camera (Canon EOS 70D), and a standard color chart (X-rite ColorChecker passport) was placed beside each individual to adjust the hue and lightness values. Bird care and all fieldwork procedures were approved by Kyung Hee University Animal Ethics Committee, and were performed in accordance with relevant national and international guidelines and regulations (e.g., catching permit, size measurement). Captured birds were safely released at the site where they were captured after data collection.

### Iris fleck scoring

**Figure 6:**
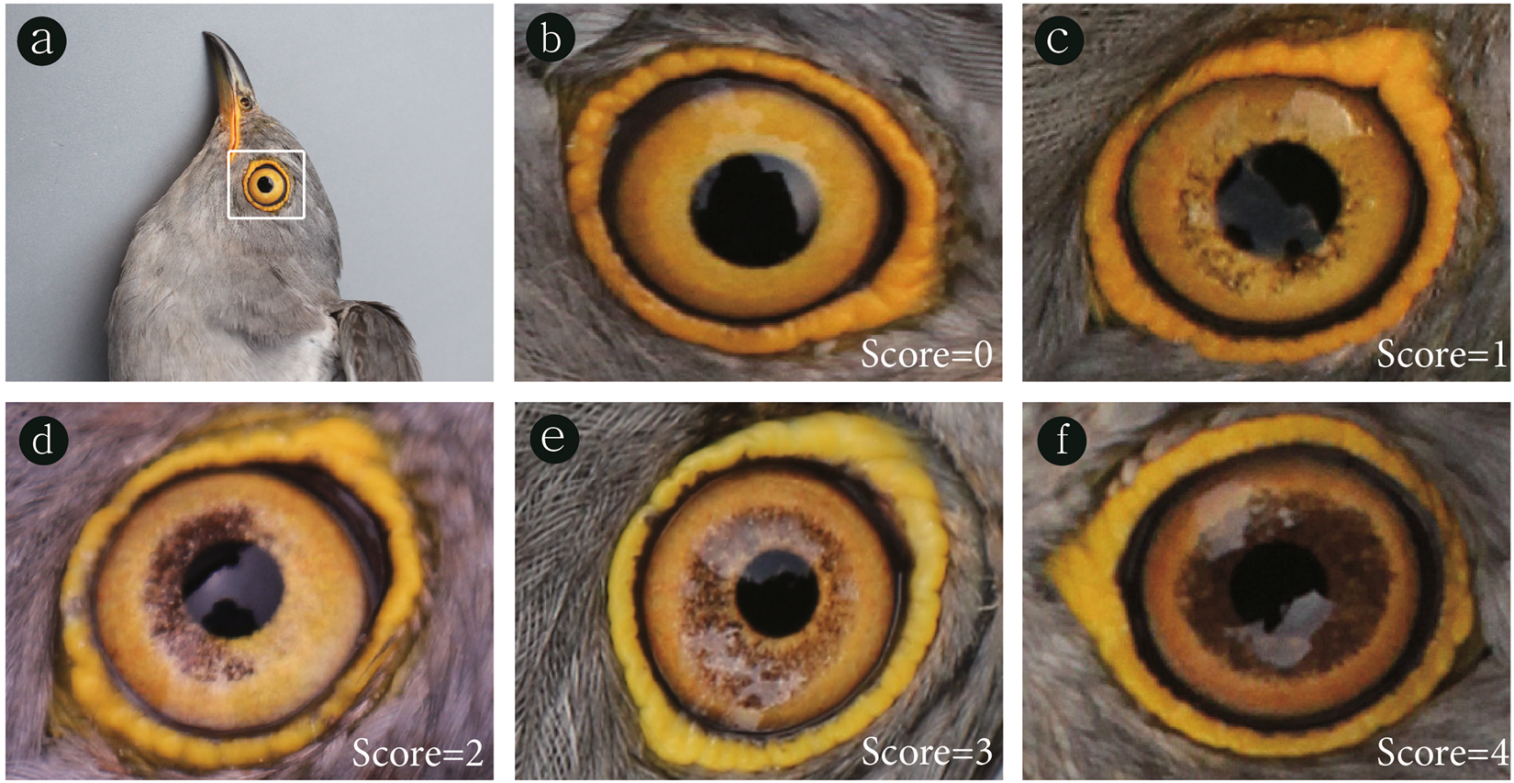
A reference standard used in this study for the amount of iris flecking in the common cuckoo (**a**). A score of 0 (**b**) was given for the iris that is clear without any flecks. In contrast, a score of 4 (**f**) was assigned when the pupil was completely surrounded by the thick band of dark flecks. Other scores of 1 (**c**), 2 (**d**), and 3 (**e**) were given according to the relative amount of flecking.

We quantified the amount of iris flecking on both sides of the eyes for 76 common cuckoos that were photographed in the field. To apply the same criteria during quantifying iris flecking, we prepared a reference standard to classify the amount of iris flecking with a semi-continuous ordinal scale (whole numbers), as shown in other studies that quantify variation in color^53–55^. Thus, 5 different categories were generated for simplicity (Fig. 6). A score of 0 was given for a completely yellow iris without any dark flecks, whereas the maximum score of 4 was given when the pupil was completely surrounded by dark flecks. Other values were assigned based on the intermediate amount (e.g., around 25%, 50%, and 75%) of iris flecking. Therefore, higher scores indicate more iris flecking, and thus, darker eyes.

Based on the reference standard, 27 people, who voluntarily participated in scoring, quantified the amount of iris flecking present in the photographs of the eyes independently. The photographs were prepared in a specific way. For each individual, we first adjusted brightness of the photo with the colour chart and cropped both sides of each eye (as in Fig. 6a) from the original photographs of the face taken in the field. Then we color-printed the photograph of each cropped eye, cut it into a rectangular shape (as in Fig. 6). We grouped the eye photographs together at random, and allowed people to score each eye against the standard. The identity of each photograph was coded, so that it could not be recognized during scoring. We also added 77 extra, duplicated photographs for 50 individuals to the set to test how people consistently scored the same photograph, which was found to be highly consistent (Repeatability = 0.97^56^). The average scores for each eye were used in the subsequent analyses. The total amount of iris flecking of an individual (hereafter termed “total iris flecking”) indicates the sum of the average iris flecking scores of the left and right eye (range: 0~8). The asymmetry in iris flecking between the left and right eye (hereafter termed “iris flecking asymmetry”) was calculated as the absolute difference between the average score of the two eyes.

### Statistical analysis

We used a Pearson product-moment correlation test (*r*) or Spearman’s rank correlation test (*r*_*s*_) to analyze correlative variation between any 2 variables, depending on the normality of the data. Likewise, any difference was tested using a *t*-test for parametric data and Wilcoxon rank sum test or Kruskal-Wallis test for nonparametric data. The effect of sex and body size on the amount of iris flecking was analyzed using a general linear model, in which sex, residual wing length as an indicator of body size^57^ and their interaction term were included as explanatory variables. We used a linear regression model to examine the relationship between total iris flecking and iris flecking asymmetry, in which total iris flecking and its quadratic term were included as explanatory variables. We also examined the relationship between iris flecking asymmetry, total iris flecking, and residual wing length using a linear regression model, in which wing length was a response variable, while iris flecking asymmetry, total iris flecking and their interaction term were included as explanatory variables. The models were validated by visual inspection of residual plots, which showed no obvious deviation from normality and homoscedasticity. The mean values are presented with standard deviation and the coefficient values of the models are presented with 95% confidence intervals (95% CI) throughout. All statistical tests were conducted in R 3.1.2^58^.
